# EXPLORING HEALTHCARE WORKERS' GERIATRIC EDUCATION AND SUBSEQUENT COMMUNICATION WITH OLDER ADULTS

**DOI:** 10.1093/geroni/igac059.1842

**Published:** 2022-12-20

**Authors:** Tina McQuaid

**Affiliations:** University of Guelph, Mississauga, Ontario, Canada

## Abstract

Research shows that older adults are living longer than ever, are the fastest growing population, and can have increasingly complex health-related issues. However, the health knowledge and literacy of older adults can be limited, and these adults may have difficulty understanding the terminology that healthcare workers use to communicate with them about their health. For impoverished older adults especially, this can contribute to poor health decisions and decreased care. Given this, educating healthcare practitioners to communicate effectively with older adults becomes essential to older patients’ quality of care. Using predominantly North American studies of healthcare workers’ practices, education, and their interactions with older adults (aged 65-85, primarily), this review paper finds that: i) older adults are responsible for their communication with healthcare workers, but practitioners, because of their implied authority, control the narrative, and therefore it is necessary for them to become more educated in communicating with older adults; ii) some current communication practices by healthcare workers (with older adults) are not reflective of sufficient care; and iii) new gerontology education can foster increased empathy and shared communication practices among healthcare workers, and this can aid patients to better control and have confidence in their healthcare decisions. Social and cultural factors that may explain the health literacy divide in older adults are discussed, as are recommendations and best practices for healthcare workers working with older adults.

